# Single agent and combination studies of pralatrexate and molecular correlates of sensitivity

**DOI:** 10.1038/sj.bjc.6606063

**Published:** 2010-12-21

**Authors:** M Serova, I Bieche, M-P Sablin, G J Pronk, M Vidaud, E Cvitkovic, S Faivre, E Raymond

**Affiliations:** 1 INSERM U728, RayLab, and Departments of Medical Oncology, Beaujon University Hospital, Assistance Publique – Hôpitaux de Paris, Paris 7 Diderot, 100 boulevard du Général Leclerc, Clichy 92110, France; 2 Laboratory of Molecular Genetics, Beaujon University Hospital, Paris 7 Diderot, Clichy, France; 3 Allos Therapeutics, Inc., Westminster, CO, USA

**Keywords:** methotrexate, antimetabolites, antifolates, drug resistance, combination chemotherapy, folate transporters

## Abstract

**Background:**

Pralatrexate is a dihydrofolate reductase (DHFR) inhibitor with high affinity for reduced folate carrier 1 (RFC-1) and folylpolyglutamate synthetase (FPGS), resulting in extensive internalization and accumulation in tumour cells. Pralatrexate is approved in the US for the treatment of relapsed or refractory peripheral T-cell lymphoma and is being investigated in various malignancies. Here, we evaluated molecular correlates of sensitivity to pralatrexate and explored combinations with a variety of anticancer agents.

**Methods:**

Antiproliferative effects of pralatrexate were evaluated in 15 human-cancer cell lines using the MTT assay. Gene expression was evaluated using qRT–PCR.

**Results:**

Pralatrexate and methotrexate had a similar pattern of cytotoxicity, pralatrexate being more potent. Pralatrexate potentiated the effects of platinum drugs, antimetabolites and EGFR inhibitors. Dose- and time-dependent cytotoxicity of pralatrexate correlated with high mRNA expression of FPGS. Acquired resistance to pralatrexate was associated with decreased RFC-1 expression, whereas methotrexate resistance correlated with increased DHFR expression, suggesting different mechanisms of acquired resistance.

**Conclusion:**

Pralatrexate was more potent than methotrexate in a panel of solid tumour lines. Our findings support the further clinical development of pralatrexate in combination with certain cytotoxics and targeted therapies, and suggest that RFC-1, FPGS and DHFR may be potential biomarkers of outcome.

The folate pathway has a key role in cell growth and proliferation (Appling, 1991; Odin *et al*, 2003). Folic acid (folate) enters cells through reduced folate carrier 1 (RFC-1), is polyglutamated by folylpolyglutamate synthetase (FPGS), and is reduced to dihydrofolate, which is further converted to tetrahydrofolate (THF) by dihydrofolate reductase (DHFR). The different enzymes and transporters involved in this pathway are targets for an important class of cytotoxic agents, antifolates. Methotrexate was one of the first agents of this class and was first used in the treatment of childhood acute lymphoblastic leukaemia (Farber *et al*, 1948). Since then, methotrexate has been widely used in haematologic and solid cancers, and new generations of antifolates have been rationally designed to exploit multiple aspects of the folate pathway (e.g., raltitrexed in colorectal cancer (Cocconi *et al*, 1998), and pemetrexed in malignant pleural mesotheliomas (Vogelzang *et al*, 2003) and non-small-cell lung carcinomas (NSCLC) (Hanna *et al*, 2004)).

Pralatrexate ((*RS*)-10-propargyl-10-deazaaminopterin) is a synthetic anti-DHFR antifolate, rationally designed to have greater affinity for RFC-1 and FPGS, resulting in increased cytotoxic activity as compared with methotrexate (DeGraw *et al*, 1993; Sirotnak *et al*, 1998). The cytotoxicity of pralatrexate was shown to be correlated with RFC-1 mRNA expression in human lymphoma cells (Wang *et al*, 2003).

Pralatrexate is undergoing clinical evaluation as a single agent or in combination in several tumour types, including lymphoma (O’Connor *et al*, 2007; Marneros *et al*, 2009), and has received accelerated approval by the US Food and Drug Administration (FDA) for patients with refractory or relapsed peripheral T-cell lymphoma.

In this study, we have compared the antiproliferative activity of pralatrexate with that of methotrexate and other antimetabolites in several human solid tumour cell lines. In addition, we further evaluated molecular mechanisms of action of pralatrexate, and screened for markers of sensitivity and acquired resistance to pralatrexate, knowledge of which could be of potential use for designing future clinical trials. Finally, possible sequence-dependent synergy or additive effects of pralatrexate with various cytotoxic and targeted agents were also investigated.

## Materials and methods

### Cell lines

A panel of colon (HT29, HCT116, COLO205 and HCC2998), breast (MCF7), melanoma (MDA-MB-435, formerly designated as a breast cancer line), NSCLC (HOP62 (adenocarcinoma) and HOP92 (large cell carcinoma)), ovarian (OVCAR3, IGROV1), prostate (DU145, PC3) and head and neck (SCC61, HEP2 and SQ20B) cancer cell lines was purchased from the ATCC (Rockville, MD, USA) and National Cancer Institute collections. Cells were grown as monolayers in RPMI medium supplemented with 10% fetal calf serum (Invitrogen, Cergy-Pontoise, France), 2 mM glutamine, 100 U ml^−1^ penicillin and 100 *μ*M ml^−1^ streptomycin. In this study, we used unpurified media potentially containing glycine, hypoxanthine and thymidine, which may have theoretically reduced drug activity. However, considering the high levels of resistance developed in our cell lines and taking into account that both parental and derived resistant counterpart were grown in similar media, it remains unlikely that this would have severely impact on data.

### Cytotoxicity assays

Cell viability was determined using the MTT assay, which was carried out as described previously (Hansen *et al*, 1989). Briefly, cells were seeded in 96-well plates at a density of 2 × 10^3^ cells per well. Cells were incubated for 120 h and then 0.4 mg ml^−1^ of MTT dye (3-(4, 5-dimethylthiazol-2-yl)-2, 5-diphenyltetrazolium bromide; Sigma, Saint-Quentin Fallavier, France), was added for 4 h at 37°C. Media was removed and the monolayer suspended in 0.1 ml of DMSO, after which the absorbance at 560 nm was measured using a microplate reader (Thermo, Saint-Herblain, France). The control value corresponding to untreated cells was defined as 100% and the viability of treated samples was expressed as a percentage of the control. IC_50_ values were determined as concentrations that reduced cell viability by 50%.

For single agent studies, cells were treated with increasing concentrations of pralatrexate, methotrexate, pemetrexed, 5-FU and 5′-DFUR for up to 72 h. Then, the cells were allowed to recover in compound-free medium for 48 h before determination of growth inhibition using the MTT assay.

For combination studies of pralatrexate with other anticancer drugs, sequential (pralatrexate for 24 h, followed by the second agent for 24 h or the inverse schedule) or simultaneous (exposure to both agents for 24 h) schedules were used. Cells were treated with various concentrations of the drugs and the combinations. Growth inhibition was then determined by the MTT assay.

### Statistical analysis and determination of synergistic activity

Drug combination effects were determined using the Chou and Talalay method (Chou and Talalay, 1984) based on the median effect principle. Combination index (CI) values of <1 indicate synergy, a CI value of 1 indicates additive effects and a CI value of >1 indicates antagonism. Data were analysed using the concentration-effect analysis software (Biosoft, Cambridge, UK). For statistical analysis and graphs, the Instat and Prism software (GraphPad, San Diego, CA, USA) were used. Experiments were performed three times, in duplicate. Means and standard deviations were compared using the Student's *t*-test (two-sided *P*-value).

### Cell cycle analysis and apoptosis

The cell cycle stage and percentage of apoptotic cells were assessed by flow cytometry. In brief, cells were treated with various concentrations of pralatrexate, fixed in 70% ethanol and stored at −20°C until use. Cells were rehydrated in PBS, incubated for 30 min at 37°C with 250 *μ*g ml^−1^ RNAse A and 20 min at 4°C with 50 *μ*g ml^−1^ propidium iodide in the dark. The cell cycle distribution and percentage of apoptotic cells were determined with a FACScan flow cytometer and analyzed by FACS Calibur (Becton Dickinson, Le Pont de Claix, France). Apoptosis induction was evaluated using an Annexin V-FITC Apoptosis Detection Kit (BD Biosciences, Le Pont de Claix, France).

### Gene expression analysis by RT–PCR

The theoretical and practical aspects of quantitative real-time PCR (qRT–PCR) using the ABI Prism 7900 Sequence Detection System (Perkin-Elmer Applied Biosystems, Foster City, CA, USA) have been described in detail elsewhere (Bièche *et al*, 2001). Results were expressed as fold differences in target gene expression relative to the *TBP* gene (an endogenous RNA control) and relative to a calibrator (1 × sample), consisting of the cell line sample from our tested series that contained the smallest amount of target gene mRNA. Experiments were performed in duplicate.

### Western blot analysis

Cells were lysed in buffer containing 50 mM HEPES (pH 7.6), 150 mM NaCl, 1% Triton X-100, 2 mM sodium vanadate, 100 mM NaF and 0.4 mg ml^−1^ phenylmethylsulfonyl fluoride. Equal amounts of protein (20–50 *μ*g per lane) were subjected to SDS–PAGE and transferred to nitrocellulose membranes. Membranes were probed with anti-cleaved PARP, anti-cleaved caspase 3, anti-caspase 9 (Cell Signaling, Saint Quentin Yvelines, France), anti-DHFR (Abcam, Paris, France), anti-*β*-actin (Sigma-Aldrich, Saint-Quentin Fallavier, France)-specific primary antibodies, followed by peroxidase-linked secondary antibodies and visualisation by chemiluminescence.

## Results

### Single-agent antiproliferative effects

**Figure 1:**
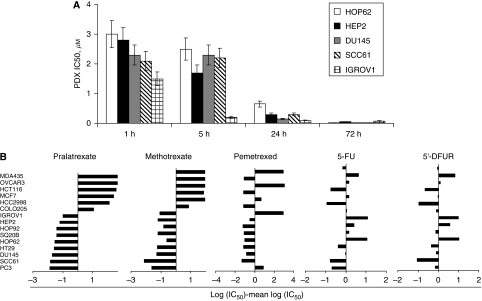
Pralatrexate cytotoxicity in a panel of human cancer cell lines. (**A**) Pralatrexate (PDX) time-course (IC_50_ values, 1, 5, 24 and 72 h drug exposure) cytotoxicity in sensitive cell lines. (**B**) Comparative analysis of 72 h cytotoxicity of pralatrexate, methotrexate, pemetrexed, 5-FU and 5′-DFUR in a panel of cancer cell lines. The indicated values are calculated as follows: log (IC_50_ individual cell line) – mean (log IC_50_). Negative values indicate that the cell line is more sensitive than the average, whereas positive values indicate that the cell line is more resistant than the average.

The antiproliferative effects of pralatrexate were examined in 15 cancer cell lines as displayed in Table 1. Time course experiments showed that optimal antiproliferative effects were achieved when cells were exposed to pralatrexate for 72 h (Figure 1A). Pralatrexate IC_50_ values ranged from 0.01±0.002 *μ*M for the prostate cancer cell line PC3 to >350 *μ*M for the MDA-MB-435 cell line. Interestingly, two groups of cell lines with more than 100-fold difference in IC_50_ were observed: One group including PC3, SCC61, DU145, HT29, HOP62, SQ20B, HOP92, HEP2 and IGROV1 cells displayed IC_50_ <0.1 *μ*M, whereas Colo205, HCC2998, MCF7, HCT116, OVCAR3 and MDA-MB-435 cells showed IC_50_ values ⩾9 *μ*M.

The antiproliferative effects of pralatrexate were compared with those of methotrexate and several commonly used antimetabolites such as pemetrexed, 5-FU and 5′-DFUR, the active capecitabine metabolite (Figure 1B and Table 1). Pralatrexate IC_50_ values were on average almost 10-fold lower than those observed for methotrexate. The cytotoxicity profiles of these two antifolates were similar with the same distinct groups of sensitive and resistant cell lines. The cytotoxicity profile of pralatrexate was different from that of 5-FU, 5′-DFUR and pemetrexed, suggesting differences in the metabolism, mechanism of action and/or resistance between pralatrexate and other antimetabolites. Interestingly, limited cross-sensitivity was observed between pralatrexate and pemetrexed, an antifolate, believed to be primarily a thymidylate synthetase (TS) inhibitor.

### Effect of pralatrexate and methotrexate on cell cycle changes and apoptosis

**Figure 2:**
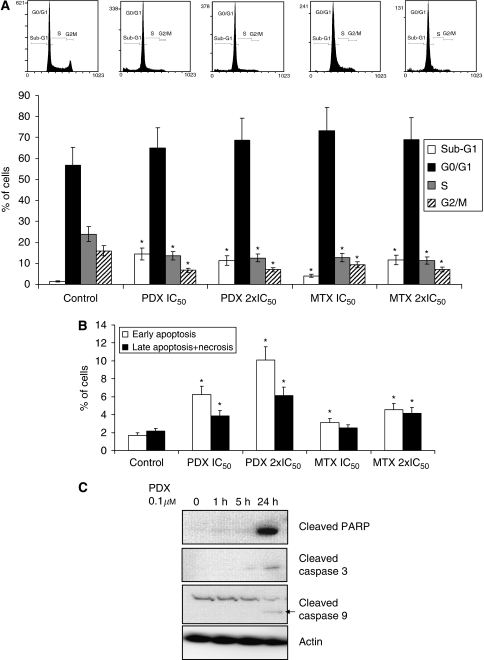
Effects of pralatrexate (PDX) and methotrexate (MTX) (24 h exposure) on cell cycle distribution (**A**), apoptosis induction (Annexin V staining) (**B**) and activation of PARP, caspase 3 and caspase 9 (**C**) in DU145 prostate cancer cells. ^*^Significant difference (*P*<0.05) comparing with control.

The prostate cancer cell line DU 145 was selected as a sensitive model to both pralatrexate and methotrexate for further investigation in this study. Cells were incubated with concentrations of 0.1 *μ*M (IC_50_) and 0.2 *μ*M (two-fold IC_50_) of pralatrexate and 0.6 *μ*M (IC_50_) and 1.2 *μ*M (two-fold IC_50_) of methotrexate for 24 h. Cell cycle analysis showed that pralatrexate-treated cells had decreased proportions of cells in S and G2/M phases with an increase of sub-G1 fraction (>eight-fold), suggesting apoptosis induction (Figure 2A). A similar pattern, albeit less pronounced, was observed in methotrexate-treated cells (Figure 2A). Thus, both agents caused the accumulation in the G0/G1 phase and possible apoptosis induction.

Annexin V/PI double staining showed that apoptosis increased 2.6- and 4-fold in DU145 cells at concentrations of 0.1 and 0.2 *μ*M pralatrexate, respectively. Similar results, with 1.5- and 2-fold increase in apoptosis, were observed in DU145 cells treated with 0.6 and 1.2 *μ*M methotrexate, respectively, again showing superior activity of pralatrexate compared with methotrexate. Apoptosis induction by pralatrexate was further confirmed by increases in cleaved PARP and caspases observed after 24 h exposure to pralatrexate (see Figure 2C). From these experiments it appears that apoptosis is the major mechanism of cancer cell death induced by pralatrexate in this model.

### Expression of genes involved in folate transport and metabolism

**Figure 3:**
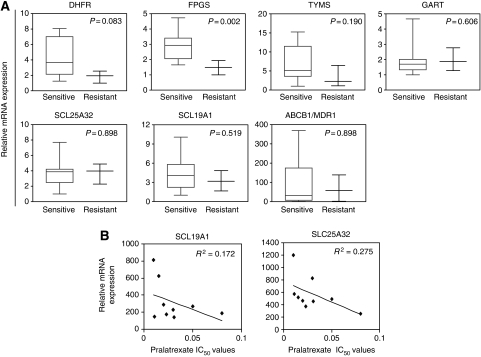
Relative mRNA expression of folate genes in pralatrexate sensitive and -resistant cell lines. (**A**) Relative expression of DHFR, FPGS, TS, GART, SLC25A32, SCL19A1/RFC-1 and ABCB1/MDR1 mRNA in sensitive- and resistant groups. (**B**) Correlation between pralatrexate sensitivity (IC_50_ values) and mRNA expression of SCL19A1/RFC-1 and SLC25A32 folate transporters in nine pralatrexate-sensitive cell lines.

The expression of genes known to be involved in sensitivity to antifolates was analyzed in the panel of cancer cell lines. DHFR, FPGS, TS/TYMS, thymidylate synthetase, SCL19A1/RFC-1, GARFT (glycinamide ribonucleotide formyl transferase), SLC25A32 (mitochondrial folate transporter/carrier) and ABC transporter B1 (ABCB1 or MDR1) mRNA expression was determined by qRT–PCR (Figure 3A). The cell lines expressed various levels of these folate pathway genes but no significant correlation was found between sensitivity to pralatrexate and mRNA expression of TS, SCL19A1/RFC-1, GARFT, SLC25A32 and MDR1. Pralatrexate-sensitive cells expressed relatively higher levels of DHFR, a target of pralatrexate, than the ‘resistant’ group, but this did not reach statistical significance (*P*=0.083, Figure 3A). Pralatrexate-sensitive cells expressed significantly higher levels of FPGS mRNA than resistant cells (*t*-test, *P*=0.002). Overall, a trend towards a positive correlation between FPGS mRNA expression and pralatrexate sensitivity (IC_50_ values) was found (*R*^2^=0.47, *P*<0.01), suggesting an important role of polyglutamation in pralatrexate antiproliferative activity.

To determine the potential role of folate transporters in pralatrexate activity, we correlated the IC_50_ values obtained after 72 h drug exposure with the level of mRNA expression of SCL19A1/RFC-1 and SLC25A32 in the nine pralatrexate-sensitive cell lines (Figure 3B). Cells that expressed a high level of SCL19A1/RFC-1 and SLC25A32 mRNA displayed higher sensitivity to pralatrexate, suggesting potential roles of SCL19A1/RFC-1 and SLC25A32 in cellular uptake of pralatrexate.

### Development of pralatrexate and methotrexate resistant cell lines

**Figure 4:**
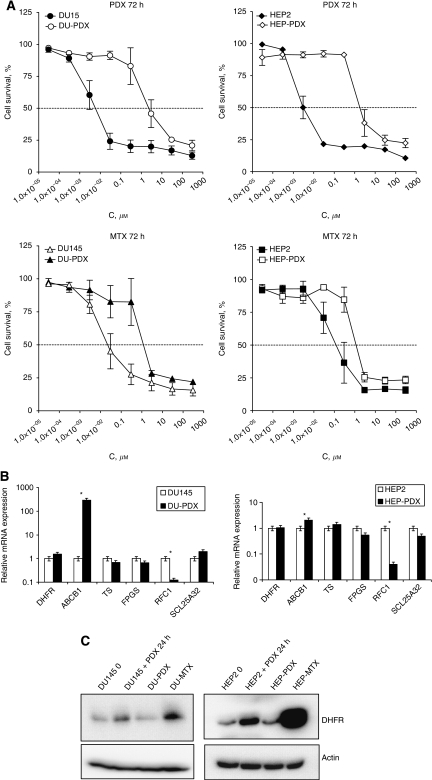
Characterization of pralatrexate-resistant cell lines. (**A**) Pralatrexate (top panel) and methotrexate (bottom panel) cytotoxicity in DU-PDX and HEP-PDX cell lines compared with their parental counterparts DU145 and HEP2. (**B**) Relative mRNA expression of folate genes in pralatrexate-resistant cell lines. (**C**) Western blot of DHFR protein in DU145 and HEP2 sensitive and DU-PDX, DU-MTX, HEP-PDX and HEP-MTX pralatrexate and methotrexate-resistant cell lines.

To characterize potential mechanisms of resistance to pralatrexate, the cell lines DU-PDX and HEP-PDX were developed from parental DU145 and HEP2 cells, respectively, by exposure to increasing concentrations of pralatrexate over a period of 6 months. DU-PDX and HEP-PDX cells were at least 200- and 500-fold less sensitive to pralatrexate than parental cells (Figure 4A). After five passages in drug-free medium, the resistant cells retained their drug resistance, suggesting stability of these cell lines. As shown in Figure 4A, pralatrexate-resistant cell lines showed partial cross-resistance to methotrexate.

To compare the mechanisms of pralatrexate and methotrexate resistance, methotrexate-resistant cell lines DU-MTX and HEP-MTX were developed. DU-MTX and HEP-MTX displayed cross-resistance to pralatrexate, however, the activity of pralatrexate still remained superior (approximately 10-fold lower IC_50_) to that of methotrexate (Data not shown).

### Genetic changes associated with acquired pralatrexate resistance

To determine possible mechanisms of anti-folate resistance, we evaluated the mRNA expression of several folate genes in parental and resistant cells. As shown in Figure 4B, mRNA expression of DHFR, TS and SLC25A32 was not significantly changed in pralatrexate-resistant cells. A slight decrease in FPGS mRNA expression was observed in DU-PDX and HEP-PDX cells compared with their parental counterparts. In contrast, SCL19A1/RFC-1 mRNA expression was >10-fold decreased in the two pralatrexate-resistant cell lines. mRNA level of ABCB1/MDR1 was 40- and 2-fold higher in DU-PDX and HEP-PDX, respectively, compared with DU145 and HEP2. These data suggest an important role of transporters in pralatrexate antiproliferative activity and acquired resistance. To study the role of MDR1 in pralatrexate resistance, DU-PDX and HEP-PDX cells were incubated with 30 *μ*M verapamil, a competitive substrate of MDR1, and 3 *μ*M cyclosporin A concomitantly with pralatrexate for 72 h. No changes were observed in pralatrexate cytotoxicity with and without verapamil and cyclosporine A, suggesting that MDR1 overexpression may not have a major role in acquired resistance to pralatrexate in these cell lines.

Analysis of expression of DHFR, a target of pralatrexate and methotrexate, showed significant increases in mRNA (more that 30-fold), as well as >10-fold increases in DNA gene copy numbers. DHFR protein expression in HEP-MTX cells was consistently higher compared with parental HEP2 cells (Figure 4C), suggesting that DHFR amplification has an important role in resistance to methotrexate (Figure 4C). Slight increase of DHFR expression in DU-PDX and HEP-PDX was nonsignificant as compared with that in parental DU145 and HEP2 cells (*P*=0.083). This suggested that the molecular mechanism of acquired resistance to pralatrexate in HEP-PDX cells might differ from acquired methotrexate resistance in HEP-MTX cells.

### Analysis of cross-resistance to other antifolates and antimetabolites

To evaluate the cross-resistance of pralatrexate-resistant cells to other drugs, DU145, DU-PDX, HEP2 and HEP-PDX cells were exposed to pemetrexed and 5-FU for 72 h. No significant difference between parental and PDX-resistant cells was observed for 5-FU cytotoxicity. Pemetrexed exposure for 72 h was only slightly less cytotoxic in DU-PDX and HEP-PDX cells compared with their parental counterparts (data not shown). These data suggest that acquired resistance to pralatrexate may not translate into resistance to pemetrexed and 5-FU, possibly because of the differences in mechanism of action of these compounds.

### Combination of pralatrexate with anticancer drugs

The effects of sequential and simultaneous exposure of combinations of pralatrexate with oxaliplatin, cisplatin, 5-FU, 5′-DFUR, SN38, paclitaxel, gemcitabine, erlotinib or lapatinib were evaluated in DU145 cells (Table 2).

The combination of pralatrexate with oxaliplatin or cisplatin resulted in synergistic effects (CI<1) when drugs were given simultaneously irrespective of the concentrations used. Pralatrexate given before oxaliplatin demonstrated only additive effects and was synergistic with cisplatin for the same administration schedule. Administration of either of the platinum drugs before pralatrexate was not beneficial. Thus, the effects of pralatrexate in combination with platinum drugs appear to be schedule dependent.

The combination of pralatrexate with the antimetabolites 5-FU or 5′-DFUR resulted in synergistic effects for the sequential administration schedules and appears to be antagonistic when the drugs were administered simultaneously. Combination of pralatrexate with SN38 (the active metabolite of irinotecan) in DU145 cells resulted in synergistic effects for all schedules of administration. The combination of pralatrexate with paclitaxel resulted in some synergistic effects for the sequential administration schedule when paclitaxel was given before pralatrexate and additive/antagonistic for the reverse sequence and simultaneous exposure. The combinations of pralatrexate with erlotinib or lapatinib were shown to be synergistic when pralatrexate was given first or simultaneously with these EGFR kinase inhibitors, suggesting a role of EGFR signalling in response to pralatrexate.

## Discussion

Pralatrexate is an antifolate with high affinity for the reduced folate carrier 1 (RFC-1) protein and folylpolyglutamate synthetase (FPGS), resulting in extensive internalization and accumulation within tumour cells. Pralatrexate is currently being investigated as a single agent and in combinations in various malignancies. In order to guide further clinical development, molecular correlates of sensitivity to pralatrexate and preclinical data on combination treatments are needed.

Pralatrexate displayed potent antiproliferative activity (IC_50_ <0.1 *μ*M) in 9 out of the 15 human solid-tumour cell lines. Two distinct groups of cell lines were identified with >100-fold difference in pralatrexate IC_50_ values: sensitive and relatively resistant cell lines. The *in vitro* antiproliferative effects of pralatrexate in terms of IC_50_ values were on average almost 10-fold better than those observed with methotrexate. When comparing the cytotoxic activity of these two similar antifolates to other antimetabolites including 5-FU, 5′-DFUR and pemetrexed, pralatrexate appears to retain activity in several cells that were poorly sensitive to 5-FU and 5′-DFUR, such as NSCLC HOP62 and HOP92 cell lines. Similarly, the sensitivity profile for pemetrexed was different from that for pralatrexate, which may be explained by the differences in molecular mechanism of action of these compounds.

Izbicka *et al* (2009) have shown that pralatrexate demonstrated unique attributes relative to methotrexate and pemetrexed. Pralatrexate exhibited enhanced cellular uptake and increased polyglutamation, which correlated with increased tumour-growth inhibition in a NSCLC xenograft model. A positive correlation between pralatrexate sensitivity and mRNA expression of FPGS, a major enzyme responsible for polyglutamation of antifolates, was found in the 15 cancer cell lines, suggesting an important role of polyglutamation in cellular response to pralatrexate. It was shown (Chabner *et al*, 1985) that if drug exposure is limited to brief periods (24 h or less), cell lines that do not form polyglutamates efficiently are insensitive to antifolate drugs. These short exposure conditions approximate clinical chemotherapy exposure and emphasize the importance of rapid production of the polyglutamated metabolites for cytotoxicity. Continuous exposure to drug may diminish the importance of polyglutamation. In our study, short (1 and 5 h) pralatrexate exposure induced pronounced antiproliferative effects and these effects may be due to improved pralatrexate cellular uptake and polyglutamation by FPGS. Cellular uptake is likely dependent on expression of folate carriers such as RFC-1 and SLC25A32. Although a positive correlation between pralatrexate sensitivity and mRNA expression of FPGS, was found in the panel of 15 cancer cell lines, only a slight but nonsignificant decrease in FPGS mRNA expression was observed in DU-PDX and HEP-PDX cells with acquired resistance to pralatrexate compared with their parental counterparts suggesting that polyglutamation may not be regarded as a main predictive factor of pralatrexate activity .

To better elucidate the predictive genetic factors of pralatrexate sensitivity, two cell lines with acquired resistance to the drug were developed from DU145 (prostate) and HEP2 (head and neck) cancer cell lines. Being more than 200-fold more resistant to pralatrexate than parental cells, DU-PDX and HEP-PDX displayed partial cross-resistance to methotrexate. Pralatrexate acquired resistance was associated with decreased RFC-1 expression and increased MDR1 expression. Fotoohi *et al* (2009) described antifolate-resistant leukaemia lines with mRNA levels of RFC-1 downregulated more than two-fold in methotrexate-resistant cells, emphasizing the important role of inux transport in antifolate resistance. Similar data were previously obtained by Jansen and Pieters (1998), Rothem *et al* (2004), and Ifergan *et al* (2003) using other methotrexate-resistant cellular models. Increased MDR1 expression does not appear to have a role in the observed acquired resistance to pralatrexate as inhibition of MDR1 did not restore sensitivity to pralatrexate. Decreased FPGS activity was shown to be associated with acquired resistance to methotrexate in human leukaemia CCRF-CEM cells (Mauritz *et al*, 2002). In our study, a slight decrease in FPGS expression was observed in pralatrexate-resistant cells, suggestive of a role for polyglutamation in resistance to pralatrexate. Studies performed three decades ago discovered that another frequent mechanism of acquired methotrexate resistance is *DHFR* gene amplification and the consequent enzyme overexpression (reviewed by Chen *et al*, 1995; Assaraf, 2007). Indeed, in our study, the cell line HEP-MTX, with acquired resistance to methotrexate, displayed a dramatic increase in DHFR mRNA and protein expression as compared with its parental counterpart. Increased DHFR expression was not observed in the pralatrexate-resistant cell lines. Although differences were not statistically significant, pralatrexate-sensitive cells expressed relatively higher levels of DHFR, a putative target of pralatrexate, than resistant cells. These data suggest that expression of DHFR may not be a major factor in pralatrexate sensitivity. These findings suggest different molecular mechanisms of resistance to methotrexate and pralatrexate in these cell lines.

The value of antifolates as anticancer treatments is potentiated by their use in drug combinations. A study of pralatrexate administration in combination with gemcitabine in a panel of lymphoma cell lines demonstrated not only that this combination is synergistic and more efficient than methotrexate/gemcitabine in generating apoptosis but also that the effects were highly sequence dependent (Toner *et al*, 2006). Pralatrexate has also been administered in combination with taxanes in a phase-I clinical trial, resulting in significant antitumour activity (Azzoli *et al*, 2007). To provide further insight into the potential clinical use of pralatrexate in patients with cancer, we combined pralatrexate with several classical anticancer drugs including cisplatin, oxaliplatin, 5-FU, 5′-DFUR, gemcitabine, paclitaxel, SN38 and the EGFR tyrosine kinase inhibitors erlotinib and lapatinib. Pralatrexate demonstrated synergistic effects with several anticancer agents and these effects were schedule dependent. Highly synergistic effects were observed by Toner, when pralatrexate was administered before gemcitabine in lymphoma models (Toner *et al*, 2006). In our experiments, simultaneous exposure to pralatrexate with gemcitabine was one of the best schedules. However, these results may be model dependent. Interestingly, our results also displayed synergistic antiproliferative effects when pralatrexate was given before or simultaneously with the EGFR inhibitor erlotinib, or the dual EGFR/HER2 inhibitor lapatinib.

In summary, pralatrexate was shown to be more potent than other antifolates in a panel of solid tumour models. Several genetic factors including RFC-1, FPGS and DHFR may be regarded as predictive factors of pralatrexate activity in solid tumour cells. Pralatrexate showed synergistic cytotoxic activity with several classical cytotoxic agents as well as with targeted EGFR inhibitors in the DU-145 prostate cancer line, and further investigation in clinical trials is warranted.

## Figures and Tables

**Table 1:** Cytotoxicity (IC_50_ values, μM) following 72 h exposure to pralatrexate, methotrexate, 5-FU, 5′-DFUR or pemetrexed in a panel of human carcinoma cell lines

**Cell line^a^**	**Pralatrexate**	**Methotrexate**	**Pemetrexed**	**5-FU**	**5′-DFUR**
PC3	0.01	0.1	2.7	1.5	25
SCC61	0.011	0.03	0.015	1.2	3.2
DU145	0.015	0.3	0.048	7	28
HT29	0.02	0.22	0.023	3	16
HOP62	0.023	0.15	0.029	78	380
SQ20B	0.03	0.26	0.025	10	27
HOP92	0.031	0.6	0.02	18	135
HEP2	0.05	0.25	0.1	86	250
IGROV1	0.08	0.33	300	8	29
COLO205	9	30	0.024	0.8	3.9
HCC2998	100	>350	1.5	10	34
MCF7	200	300	0.022	1.3	7.8
HCT116	280	>350	350	10	45
OVCAR3	>350	>350	0.025	31	230
MDA435	>350	>350	300	5	33

a Cell lines used: colon (HT29, HCT116, COLO205 and HCC2998), breast (MCF7), melanoma (MDA-MB-435, formerly designated as a breast cancer line), NSCLC (HOP62 and HOP92), ovarian (OVCAR3 and IGROV1), prostate (DU145 and PC3), and head and neck (SCC61, HEP2 and SQ20B).

**Table 2:** Effects of combinations of pralatrexate with other anticancer drugs in DU145 prostate carcinoma cells

	**Combination index**
**Combination with**	**median (95% confidence interval)**
*Oxaliplatin*	
Pralatrexate–oxaliplatin	1.03 (0.66–1.54)
Oxaliplatin–pralatrexate	1.61 (1.33–2.19)
Pralatrexate+oxaliplatin	0.66 (0.61–0.74)
	
*Cisplatin*	
Pralatrexate–cisplatin	0.66 (0.50–1.10)
Cisplatin–pralatrexate	1.12 (1.00–1.34)
Pralatrexate+cisplatin	0.68 (0.42–1.28)
	
*5-FU*	
Pralatrexate–5-FU	0.81 (0.58–1.19)
5-FU–pralatrexate	0.87 (0.69–1.80)
Pralatrexate+5-FU	1.21 (0.78–1.53)
	
*5′-DFUR*	
Pralatrexate–5′-DFUR	0.69 (0.52–1.26)
5′-DFUR–pralatrexate	0.638 (0.40–1.02)
Pralatrexate+5′-DFUR	2.20 (1.42–4.21)
	
*SN38*	
Pralatrexate–SN38	0.36 (0.21–0.74)
SN38–pralatrexate	0.58 (0.45–0.76)
Pralatrexate+SN38	0.76 (0.53–0.99)
	
*Paclitaxel*	
Pralatrexate–paclitaxel	1.18 (0.86–2.02)
Paclitaxel–pralatrexate	0.75 (0.59–0.81)
Pralatrexate+paclitaxel	1.33 (1.02–1.62)
	
*Gemcitabine*	
Pralatrexate–gemcitabine	0.96 (0.56–1.34)
Gemcitabine–pralatrexate	1.01 (0.47–1.42)
Pralatrexate+gemcitabine	0.68 (0.16–1.13)
	
*Erlotinib*	
Pralatrexate–erlotinib	0.29 (0.12–0.90)
Erlotinib–pralatrexate	0.89 (0.72–1.28)
Pralatrexate+erlotinib	0.77 (0.67–0.88)
	
*Lapatinib*	
Pralatrexate–lapatinib	0.81 (0.62–1.23)
Lapatinib–pralatrexate	0.92 (0.73–1.47)
Pralatrexate+lapatinib	0.59 (0.30–0.94)

Medians (95% confidence interval) of combination index were calculated from two independent experiments. Combination index (CI) <1 indicates synergy, CI >1 antagonism, whereas a CI equal to 1 corresponds to an additive effect.
